# Community-based participatory planning contribution to social capital for enhanced disaster resilience in rural Matobo, Zimbabwe

**DOI:** 10.4102/jamba.v15i1.1409

**Published:** 2023-11-22

**Authors:** Thabo Ndlovu, Mthabisi Msimanga

**Affiliations:** 1 Institute of Development Studies, Faculty of Commerce, National University of Science and Technology, Bulawayo, Zimbabwe

**Keywords:** community-based participatory planning, CBPP, disaster resilience, social capital, ward development planning

## Abstract

The infusion of participatory methodologies in Zimbabwe gained momentum across rural and urban development planning platforms in the past decades. Participatory approaches are envisaged to deepen grassroots involvement in local planning and strengthen social networks for enhanced disaster resilience against complex weather-related hazards such as droughts. This article examines how community-based participatory planning (CBPP) contributes to the cohesiveness of the vulnerable groups and harnesses local capacities in building social capital. The article is underpinned by the community capital framework in understanding the nexus between participatory planning and social capital. Primary evidence is drawn from the cross-sectional study that targeted different socio-economic groups (*N* = 120) drawn from the selected three wards out of the 10 where CBPPs were conducted in Matobo district in 2016. The sample size was informed by the Rao Soft sample size calculator, hence a total of (*n* = 90) socioeconomic groups were targeted for a semi-structured questionnaire. The semi-structured tool was complemented by 12 in-depth interviews and three focus group discussions. Primary data were systematised, cleaned and analysed using the Statistical Package for Social Sciences (SPSS) V23. This was meant to probe and capture the benefits of CBPP and how it has strengthened the social capital of drought-prone communities in rural Matobo. Study results indicate improved inter- and intra-community networks post the CBPP processes in drought-prone Matobo as evidenced by the formation of community clubs, gender inclusive interventions and improved financial and material contributions towards community-driven projects. Furthermore, while women involvement is evident, their influence is less in productive livelihoods activities such as livestock rearing.

**Contribution:**

The CBPP process has shown that inclusive planning improves the identification of risks and strengthens collective actions towards design and implementation of resilience building strategies such as water harvesting and health centres.

## Introduction

With development and resilience building processes continuing to evolve, participatory planning approaches have dilated in recent past as researchers and practitioners seek apt ways to involve vulnerable groups in decision-making from needs identification through to implementation (Alam & Ihsan 2020). The recognition of the essence of participation in community-based planning can be traced back to the early 1950s (Nour 2011), emerging in contemporary resilience building as the valuable processes of engaging vulnerable groups in rural Africa (Chisinga 2003). In his work, Phiri (2015) corroborates that community-based approaches gained traction in post-World War II, while in other disciplines it can be traced back to the late 1960s and the 1970s (Lassa et al. 2018). Involving have-nots in resilience planning is meant to improve the quality of the decisions and further cement ties to exploit lived experiences and local knowledge (De Graaf, Van Hulst & Michels 2015). Have-nots in this context refer to vulnerable groups with limited space to express themselves. The crux of involving have-nots in resilience planning is to allow them to exercise ‘choices in the development of human, organisational and management capacity to deal with diverse shocks. Hossain (2013) corroborates that involvement of have-notes in resilience planning galvanises cohesion as the ownership feeling is entrenched and as the recognition of the benefits of their inclusion becomes apparent. Have-nots are guardians of societal risks as they work hand in hand with governments, donors and non-governmental organisations (NGOs) (Van Niekerk et al. 2018). This deepens the relevance of community-based participatory planning (CBPP) processes in allowing have-nots and other partners unpack the local context (Corburn 2005) and inform the timing of mobilising community actions (Praharaj, Han & Hawken 2017). In support, Schatz and Rogers (2016) posit that CBPP is a shift from the so-called ‘top-down’ towards strategic planning based on contributions from have-nots, while Chambers (1992) opines that it is a process meant to transform well-being.

This article examines the contribution of the CBPP processes to the social capital in rural Matobo. To achieve this, the theoretical framework underpinning the study is presented, followed by the discussion on the nexus between participatory planning and social capital as well as the methodology. Lastly, the article presents an analysis of the benefits of the CBPP processes and the social norms they build before drawing a conclusion.

## Community-based participatory planning and ward development planning in Zimbabwe

Participatory planning processes normally imply two-way exchange of views via decisional forums (Boyer-Villemaire et al. 2014) inclusive to empower diverse socio-economic groups. The process allows have-nots to collectively set yardsticks, communicate and understand the implications of their intended actions in material and financial terms. To entrench the involvement of vulnerable communities in rural Africa, the African Charter on Popular Participation was legislated in 1990 following the hosting of the United Nations Conference on Popular Participation in Arusha, Tanzania. The outcomes of this conference viewed participation as a fundamental dimension of sustainable development, as expressed in the Rio Declaration on Environment and Development (Boyer-Villemaire et al. 2014). However, the pitfall of CBPP arises when development practitioners treat vulnerable groups as monolithic and homogeneous, without accounting for diverse intelligence (Hollander 2012). Such attitudes accentuate the need for consultation with diverse groups in reaching for a consensus on a plan and its implementation.

The advent of CBPP tool under the three-pronged approach (3PA) in early 2010 to strengthen resilience planning at national and subnational levels has generated interest among local authorities in Zimbabwe. The 3PA tool comprises the Integrated Context Analysis, Seasonal Livelihood Programming and CBPP tools. The United Nations and World Food Programme developed the 3PA in consultation with governments and its partners to inform planning and devise context-specific interventions dealing with shocks. The CBPP is a community level participatory exercise that empowers vulnerable communities to build a common thought on livelihoods, landscapes, shocks and stresses, susceptibilities and priority needs to formulate multi-sectorial action plans tailored to the local context (WFP 2016). This is a 5-day consultative field exercise rolled out to develop a 3-year programme plan for a given community at subnational level. The exercise allows participants to gain more insights into obtaining household profiles, spatial layout, shocks and stressors bewildering different parts of the area as well as options available to transform living conditions. Such practices normally make the development process to be more bottom-up and people-centred than top-down, which is starkly disapproved in many academic and professional spheres (Iqbal 2018). The promotion of the CBPP was meant to complement the existing traditional Ward Development Planning processes as enshrined in the *Zimbabwe Rural Councils Act* (Chapter 29:13) of 1988. The Act provides for the establishment of Ward Development Committees normally chaired by the local councillor to spearhead local growth and preside over the formulation of ward development plans. Equally relevant is the 1984 Prime Ministerial directive that led to the formation of coordinative structures such as the Village Development Committees (VIDCOs), Ward Development Committees (WADCOs), District Development Committees (DDCOs), and Provincial Development Committees (PDCs). These development planning platforms exist to expand participation of grassroots organisations and individuals in rural Zimbabwe and to hold government and NGOs accountable (Masue & Askvik 2017).

The major thrust of subnational structures is to ensure inclusion of disadvantaged groups (women, youth and people living with disabilities) in decision-making and subsequently in local economic development. The expectation is that locally available resources such as minerals, wildlife, water bodies, agricultural activities, human capital, among others, will be accounted for, for the benefit of the local community. However, there is a perception that Ward and Village Development Planning processes negate the principles of participatory engagement and narrow consultations to a few members. The contention is that instead of ward development committees acting as a channel for bottom-up initiatives, it has become primarily the receiver of information and directives from above especially central government and at times from political party superiors. In the process, vulnerable groups are sidelined in decision-making processes that shape their future. In concurrence, Cooke and Kothari (ed. 2001) indicate that participatory planning process is a lip service, at worst becomes manipulative to participants on the pretext of information exchange. To this end, the discussions on the social gains proffered by CBPP as a resilience-planning tool have become necessary.

## Social capital and participatory planning nexus – theoretical reflection

Social capital is viewed as a complex, multidimensional concept (Shiell, Hawe & Kavanagh 2018), while in some spheres it is deemed as social glue which makes things happen (Flora & Jan 2004). Social capital has historical roots in sociology (Gannon & Roberts 2018) and is reflected by the productive value of social connections (OECD 2013). The social capital term use has origins in the work of Hanifan in 1916 (Putnam 2000) and has become fashionable in understanding relational social dynamics in strengthening the resilience during pre- and post-disaster planning phases (Ritchie & Gill 2018). The resultant social engagements normally oil the wheels of collective deeds (Adger 2001). Putnam’s work deems social capital as a positive asset comprising trust, norms and networks influential in fostering community cohesion (Coleman 1990; Putnam 2002) cited in Daykin et al. 2021. He views trust, norms, and networks as the derivatives of the existing social base. Theoretically, social capital is a vehicle for dissemination of information to individuals and group members to enhance their productivity, while Bourdieu’s accentuates that it strengthens network membership in terms of access to resources and opportunities (Gannon & Roberts 2018), which is largely influenced by the quality and quantity of networking generated prior (Iqbal 2018). Interestingly, social capital may harbour harmful norms especially where unequal distribution of power exists among individuals in the network (Iqbal 2018).

Development planning cannot afford to ignore the benefits of intra- and inter-networks (Jakobsen, Clausen & Andersen 2020) if local resources are to be fully exploited through enhanced social exchange. Social capital is categorised as bonding or bridging (Putnam 2000), with bonding inward looking and essential in strengthening ties among homogenous groups, while bridging is outward looking and focuses on diverse groupings. Participatory planning is one of the avenues through which bonding and bridging ties can be strengthened as corroborated by Straub et al. (2020) that social capital is reflected in relationships between individuals as well as goods and services transferred through these networks. Bonding capital can result in exclusively isolating members who do not espouse a fundamental identity. The study examined the existence of trust between individuals and community groups post the CBPP processes. Trust is an important component in influencing the ability to take collective action for the benefit of the community (Villalonga-Olives & Kawachi 2015).

Bearing in mind that resilience is not only a function of involvement, the Community Capitals Framework (CCF) by Flora and Flora was used to scrutinise social capitals generated post CBPP given its wide usage in community development, resilience, and planning (Stone & Nyaupane 2015). The CCF, a strategic planning tool, is essential in asset mapping and unpacking the interdependence between capitals (Anderson 2014). The framework defines a ‘capital as any type of resource capable of producing additional resources’ (Flora & Jan 2004). The CCF constitutes seven capitals (human, social, political, cultural, built, natural, and economic or financial) that as individuals and in combination can be deployed and transform community capacities to absorb shocks (Pigg et al. 2013). The CCF resonates with participatory planning for holistic analysis of assets within settings (Duffy et al. 2016) and explores the potential of capitals to contribute towards resilience building (Flora & Flora 2008). The CCF became relevant following the realisation that community capacity is ‘the summation of influence of a community’s commitment, material and financial resources and skills that can be utilised to enhance community strengths, deal with local challenges and exploit opportunities’ (Robson 2015). However, the study sought to flag how the CBPP influenced social capital in rural Matobo. The output of the CBPP processes, that is Community Action Plan sums up capacities and outlines clear indications on the resilience building intentions of the community. Without engaging communities and understanding their context, planning resilience interventions is compromised. Flora, Flora and Gasteyer (2015) concur that cataloguing community resources by recognising interdependence, interaction, and synergy fosters resilience-building efforts. It is critical to note that community planning is dynamic especially in ensuring that all members of a group contribute without compromising decision-making processes.

## Study area

Matobo district is located in Matabeleland South province covering an area of 7220 km^2^. The district constitutes 25 wards with 49% of the total area being communal, while the other 51% of the area consists of resettlement area, grazing land and game reserve area. District population is 95 696 with an average household size of 3.9 people according to the 2022 census (Zimstat 2022). The district lies in the semi-arid agro ecological regions 4 and 5, with region 6 lying on the northern part of the district. Matobo district is characterised by long dry spells and persistent droughts and receives an annual average rainfall of 350 mm – 600 mm. Semi-extensive to extensive livestock ranching, production of small grains deemed drought tolerant are common farming activities that characterise the district. Wildlife^1^ is abundant especially in areas formerly with large-scale commercial farming areas, hence incidences of human–wildlife conflict are inevitable. The southern wards (1–10) of the district experience water challenges for domestic and livestock owing to long dry spells compared with northern wards (15–25), which receive higher precipitation. The recurrent droughts have reduced safe water supply in the district as water points have dried up leaving approximately 30% of rural boreholes functional. The capacity to drill boreholes in wards in the southern parts is restricted owing to the underlying rock, which is granitic and hard to drill. This has compelled communities, especially women and girls to travel long distances in search of safe drinking water sometimes as far as 5 km from the area of residence. Communities are collecting water from nearby rivers as the alternative safe water sources are more than 5 km away (Matobo district profile 2021). Matobo district has 12 large functional dams, which service irrigation schemes such as the famous valley and Agriculture Rural Development Authority (ARDA) enabling communities to produce maize crops, winter and summer wheat and groundnuts. The mass production of these crops through irrigation farming helps in stabilising the price of grain and improves access to local communities. The main types of livestock reared in the district are cattle, goats, donkeys, sheep and chickens.

## Research methods and design

A cross-sectional research survey design was used where three study sites (wards) were selected out of 10 wards that rolled out CBPPs in 2017 in Matobo district. A convergent mixed methods approach was used to gather data in June 2022. This entailed the collection of quantitative and qualitative data concurrently. The respondents were drawn from different socio-economic groups (farmers, women clubs, widowers, business community, artisanal miners, traditional leaders, vendors), district and ward level government departments, NGOs, Rural District Council staff with direct and indirect participation in local planning processes in Matobo. The study gathered quantitative data by administering 90 semi-structured questionnaires while qualitative data was gathered from 12 in-depth interviews and 3 focus group discussions.

The study purposively sampled key informants and 3 wards out of 10 that implemented CBPPs in year 2017. Selection of the wards was premised on the accessibility based on the time and resource constraints of the research and the fact that the three wards had similar socioeconomic settings as other non-selected wards. In 2017, 40 participants drawn from different socioeconomic groups were targeted in each ward to participate in the CBPP. This translates to a target population of (*N* = 120) for all the three wards. The primary quantitative data sample sizes for the research were derived using a computer and/or web-based sample size calculator (Rao Soft) (http://www.raosoft.com/samplesize.html). The acceptable margin of error was pegged at 5% with the confidence level for sampling pegged at 95%. Respondents from the three wards were randomly selected to give each an equal chance of inclusion with sample size of 92 (*n* = 92) equally distributed across the study areas. However, because of unforeseen circumstances that included the reluctance of targeted respondents to inform the study, a final sample size of (*n* = 90) was reached giving a response rate of 97.8%. The socio-economic characteristics were the same, hence the equal distribution. The sampled were engaged to discern the benefits and experiences post CBPP processes as well as networks that accrued. The questionnaire was pretested in communities with similar characteristics but in a ward that was not part of the study. The engagement of respondents was exercised under strict adherence to coronavirus disease 2019 (COVID-19) protocols including social distancing, wearing of masks and sanitisation of hands of all involved. The study engaged local extension personnel to administer structured questionnaires following a rigorous training to enhance standardisation and uniformity in administration. Key informants drawn from government ministries at district and ward levels, NGOs, donors and traditional leadership were engaged through in-depth interviews. Focus group discussions comprising 10 socioeconomic groups not targeted using structured questionnaires were conducted in each of the three wards to examine networks that developed post CBPP in rural Matobo.

Data were organised, cleaned and analysed using the Statistical Package for Social Sciences (SPSS) V23 where different reckonings were applied to give meaning to the data. These included cross tabulations, Likert ranking scale analysis and variable reductions through principal component analysis. In the determination of social capital gains or benefits of CBPP, 11^2^ principal components were initially defined and these were reduced to five^3^ components that marked the benefits and/or capital gains. The reduction was informed by the frequency of grouped responses and/or themes, which were further aligned with the gains that evolved in the qualitative response analysis. In an effort to determine the magnitude of women empowerment, gender analysis was computed and was presented as mean ± SD with the level of significance set at *p* < 0.05 and a *t*-test was used to test for mean differences. Qualitative data were analysed using NVIVO version 12 and thematic content analysis applied.

### Ethical considerations

Permission was sought from Matobo Rural District Council to conduct the study in the three selected wards. Furthermore, the consent of participants was sought before interviews were conducted guaranteeing them observance of their rights to confidentiality throughout the study.

## Results and discussion

### Benefits of community-based participatory planning

As indicated earlier, CBPP approaches offer grassroots the opportunities to influence the planning processes and local decisions. In this regard, inclusivity becomes a necessity towards gathering relevant data for informed decisions. This makes gender considerations very significant in enhancing inclusive planning. However, despite that equal representation of women and men in planning platforms is meant to allow for the collection of diverse views, in the majority of cases, dominating in numbers does not guarantee influence in decision making. Women have, in the majority of cases, been left out or dominated in local decisions owing to cultural and other local dynamics. The results in Table 1 indicate that 53% of the respondents were female while 47% were male. There was a higher number of women than men because the majority of men are outside the area seeking alternative sources of income and/or expanding their livelihood base (Table 1, Table 2 and Table 3). This was confirmed by one of the ladies who said in ‘isindebele *obaba awubatholi ekhaya*’, meaning men rarely reside at home, a sign that they are highly mobile seeking ways to strengthen their ability to absorb shocks.

**TABLE 1:** Demographics.

Variable	%	*n*	SD	*p*
**Gender**				
Female	53.3	48	-	-
Male	46.7	42	0.502	0.000
**Socioeconomic groups**				
Famers	86.7	78	-	-
Business	8.9	8	-	-
Women clubs	21.1	19	-	-
Traditional leaders	11.1	10	-	-
**Impact of CBPP†**				
Has improved	95.6	86	-	-
No improvement	4.4	4	-	-
**Women livelihood activities‡**				
Livestock	14.4	13	-	-
Dry land cropping	75.6	68	-	-
Vending	5.6	5	-	-
Remittances	2.2	2	-	-
Irrigation farming	2.2	2	-	-

SD, standard deviation.

† , Has CBPP improved participation of socio-economic groups;

‡ , Livelihood activities in which women are involved in.

**TABLE 2:** Asset capital ranking by level of importance.

Asset capital	Extremely important	Very important	Moderately important	Neutral	Slightly important	Low importance	Least important
Livestock	40.0	37.8	10.0	4.4	1.1	1.1	5.6
Dryland cropping	58.9	30.0	3.3	3.3	1.1	1.1	2.2
Artisanal mining	7.1	1.2	2.4	3.5	32.9	16.5	36.5
Vending	9.7	1.4	16.0	38.0	11.1	28.0	20.8
Remittances	8.6	7.1	34.0	24.0	7.1	2.9	18.6
Fishing	1.4	0.0	0.0	1.4	5.7	30.0	61.4

**TABLE 3:** Level of women engagement in livelihood activity before CBPP and after.

Livelihood activity	Insignificant	Low	Not sure	Satisfactory	Very significant
**Livestock**					
Before	7	6	0	0	0
After	0	0	0	2	11
**Dry land cropping**					
Before	11	45	4	7	1
After	0	0	1	26	41
**Vending**					
Before	1	3	1	0	0
After	0	0	1	3	1
**Remittances**					
Before	0	2	0	0	0
After	0	1	0	1	0
**Irrigation farming**					
Before	0	2	0	0	0
After	0	0	0	0	2

CBPP, community-based participatory planning.

Promoting the participation of all group in community development is a non-negotiable principle of the CBPP process, hence reference to gender became inescapable. The understanding of gender informed practices such as mobility, which is anchored on cultural settings is a significant factor in building the resilience to varied shocks. Mobility deepens men’s ability to adapt to varied shocks, establish links to support coping and adaptation to varied shocks for their families. At the same time, the absence of men presents women with an opportunity to be part of the planning processes and voice their needs in shaping development pathways. The CBPP made it possible for participating men and women to contribute to the profiling of local shocks, resources and learn more about opportunities that exist to counter threats in different seasons. The study suggests that the CBPP exposed women to community planning processes and had them selected to preside over satellite committees established to oversee the implementation of the Community Action Plans. Such a trend reflects a shift from the society-wide belief that women rarely influence decision making as corroborated by Ndlovu and Mjimba (2021) that women power in areas of influence is limited. In-depth discussion revealed that women need to consult their spouses before implementing decisions they deem appropriate to address shocks and this reflects a limitation they face. In concurrence, one of the male respondents said ‘we may agree to implement interventions to deal with shocks as a group. However, women will have to consult with their spouses first and this retards collective efforts’ (Ward 8, farmer, male). This view mirrors entrenched patriarchal and cultural tendencies that suppress women and infringe on their rights to make independent decisions. The notion resonates with the CFF that capitals especially cultural, influence the voice that is heard and listened to in certain spheres (Spring, Carter & Blay-Palmer 2018). This has generational links where women particularly in the African set up have for long been viewed as incapable of making sound decisions. Through the CCF lens, strategies can be built and fortified to enhance the contributions of all socioeconomic groups in strengthening social capitals.

The findings on livelihood activities point to dry land cropping (58%) and livestock farming (40%) as the key sources of survival for the Matobo community with women participation high in dryland cropping (76%) compared with livestock farming (14%). In-depth discussion indicates the significance of remittances as one of the pillars of survival in rural Matobo. Remittances especially from neighbouring countries increase during times of distress and when schools open. Historically, men have dominated the livestock sector and it has over the years been their citadel of power. The results are consistent with the findings by Herring et al. (2018) that men are in control of livestock and that as part of tradition, they are the ‘pillars of the home’ with absolute authority to either increase or decrease the homestead’s herd of cattle. The limited involvement of women in livestock because of culture that is suppressive and weak empowerment initiatives targeting women suggests a dearth in networks in the sector, hence their influence is curtailed. However, there are isolated cases indicating an increase in women’s influence especially on small stocks such as goats and chickens. The limitations of women in participating in other spheres of livestock farming such as cattle production challenge institutions to devise creative ways of promoting inclusive farming. The proportion of women into livestock post the CBPP confirms the control men have on cattle farming with a slight shift in goat farming as corroborated by one of the respondents who said:

‘Our influence in cattle production is low, women involvement is increasing in the rearing of small stock like chickens. Following CBPP rollout, I was enlightened and today am the proud owner of 30 goats and I am helping my household in number of ways with my income, (Ward 3, livestock farmer, female)

Broadly, women’s engagement in local development has improved including dry land cropping where 98% acknowledged women dominance (38% were satisfied and 60% found dry land cropping very significant). Capital requirements in dryland farming are inevitable although not prohibitive, hence the majority of women maximise given the number of institutions offering agricultural assistance. The stringent access to capital sets back women empowerment initiatives as the majority are incapacitated to raise the required asset thresholds to benefit from credit facilities. Interestingly, women provide much labour and contribute to assets expansion at household level: an affirmation that they are a critical source skill in community development, hence their inclusion cannot be overemphasised.

Socioeconomic groups representatives constituted farmers (87%), women clubs (21%), traditional leaders (11%) and the business community (9%). In communal Matobo, dominant livelihoods are agro-based, hence the majority represented the farming community. Some respondents could not recall the groups they represented when CBPPs were conducted. It was observed that some members are involved in more than one socio-economic group owing to multiple level responsibilities that they assume in the community. The identification of socioeconomic groups is a critical component towards embracing diversity and promotion of inclusivity in championing local development. The engagement of different socioeconomic groups presents an opportunity for communities to link up and understand interdependencies and the benefits.

The CCF accentuates the importance of the interactions of different capitals, which is shaped by how varied community skills are utilised. Thus, it is essential for community planning to acknowledge existing community groups and their livelihoods to effectively identify functional links and map potential networks to strengthen community’s ability to respond to shocks. Profiling socioeconomic groups helps to unpack complex human aspects and gaps that can either promote or derail collective efforts, self-organisation when responding to local and external shocks and stressors (Apostolopoulos, Newbery & Gkartzios 2019). Exclusion of socioeconomic groups leads to omission of important dynamics, variables and essential information within a community (Mueller et al. 2020). It is paramount for the CBPP processes to ensure fair representation of the socioeconomic groups to leverage community traits to achieve set goals. The mix of vulnerable and less vulnerable socioeconomic groups inspires communities to pull together and surpass their confines.

### Community-based participatory planning and social networks

Community connections are vital in cementing intra- and inter-relations. Most importantly, networks are commonly anchored on agreed upon norms and trust. Through the networks, as indicated in Table 4a, Table 4b, Table 5, Table 6 and 7, individuals and communities leverage skills and valuable information regarding their area. Not only does this engender cohesiveness but it also improves contributions to local development and raises awareness on the local context as information flows through the linkages.

**TABLE 4a:** Social networks.

Institutions that have improved post CBPP	%	*n*	Individual benefits derived from these institutions	%	*n*
Prop confirming network development because of CBPP	100	90	Improved access to financial resources	64.4	58
VSLA groups	70.0	63	Access to food handouts	55.6	50
Livestock committees	54.4	49	Received agricultural inputs	86.7	78
Government ministries	68.9	62	Clarity of resource profile	20.0	18
Church organisations	22.2	20	-	-	-
NGOs	51.1	46	-	-	-
Traditional leadership	33.3	30	-	-	-

Note: Prop confirming network development because of CBPP (*N* = 90; 100%).

CBPP, community-based participatory planning; NGOs, non-governmental organisations; VSLA, village savings and loan associations.

**TABLE 4b:** Social networks.

Prop of community networks developed post CBPP	%	*n*	Prop of networks that have availed financial resources	%	*n*
**Community developed networks**					
Government ministries	49	53	Government ministries	48.9	44
Local development committees	64.4	58	Local development committees	38.9	35
Business community	22.2	20	Business community	13.3	12
NGOs	63.3	57	NGOs	70.0	63
UN agencies	7.8	7	UN agencies	-	-

NGOs, non-governmental organisations; CBPP, community-based participatory planning; UN, United Nations.

**TABLE 5:** Elements that have improved as a result of CBPP.

CBPP elements that have improved	%	*n*
Community cohesion	98.9	89
Community trust	92.2	83

CBPP, community-based participatory planning.

**TABLE 6:** Proportion that feels that men and women are equally benefiting.

Variable	Strongly disagree	Disagree	Neutral	Agree	Strongly agree
Are men and women equally benefiting from these networks	34.4	15.6	24.4	22.2	3.3

**TABLE 7:** Networks more beneficial to women and men.

Network	Women	Men
Government ministry	35.3	13.3
NGOs	37.9	12.2
Business community	1.3	27.8
Local development committees	16.3	2.2
Traditional institutions	9.2	7.8
Nothing	0.0	36.7

NGO, non-governmental organisations.

A very significant proportion (95.6%) of the respondents indicated that the CBPP improved the participation of different socio-economic groups in community development. The The results in Table 4 suggest that individual networks improved as more Village Savings and Loan Association (VSLA) groups were established and there were more engagements, that is, 69% with government, 51% with NGOs while 54% with local development structures such as the livestock development committees. Village Savings and Loan Association are self-managed associations which provide members with a secure means to keep their money and access it (Ksoll et al., 2015). Broadly, these are community-based financial establishments for the intent of helping members and outside members access credit. Such loans are usually accessed at low interest rates. The objective is to promote thrift, self-reliance and financial inclusion among rural communities, particularly the marginalised groups. The findings indicate that women are benefiting more from government line ministries and NGOs when compared with men resulting from empowerment models promoted by these networks. Targeting of women by these networks is encouraging. However, more needs to be performed to improve women’s influence on decision making at household and community levels as corroborated by one of the respondents that ‘women receive most of the support inputs from government and NGO but men have the final say on their use’ (Ward 8, traditional leader, male). Men’s power is concentrated more on entrepreneurship allowing them to dominate in retail shops and sole trading. The desire to be self-reliant prompted the majority of women to be part of VSLAs to enhance access to financial capital. Other benefits accruing from these individual networks include the flow of resources largely through agricultural inputs and food assistance interventions.

The improved government networks were influenced by the programmes such as the agriculture input distributions rolled out by the department of Agriculture Technical and Extension Services to support mainly dryland farming. Such initiatives by the government influenced extension services to improve on household monitoring programmes and enhance contact with target groups. Local management committees connect easily with individuals to appraise them on development initiatives and they are a useful tool in conveying important information on local development. Community networks were strongest with local development communities (64%), followed by NGOs (63%) and then government ministries (59%). Notably, the traditional leadership (33.3%) connection with the constituency did not improve much post CBPP. This is largely because of their engagement in politically related initiatives that taint their image and alter objectivity in making decisions about their constituencies. Traditional leaders are one of the institutional bodies that will always be resident in communities, hence networks with individual and communities should be strengthened.

The bridging social networks are necessary links with different groups of people and institutions to strengthen commonalities rather than differences between them (Iqbal 2018). The links make things happen by pooling together abilities and skills that are dormant and not realised and utilised. Intra- and inter-community relations (bonding and bridging social capital) have significantly strengthened the resource mobilisation drive in Matobo as evidenced by the collective construction of Nhlupho Rural Health Centre. One of the traditional leaders said:

‘It wasn’t easy, we mooted this idea long back and we have managed to pool together locally the financial and material resources towards the construction of the clinic with the support of Matobo Rural District Council.’ (Ward 10 traditional leader, male)

The implementation of Nhlupho health project drew interest from Matobo Rural District Council and has engendered feelings of belonging and shared identity. This scenario emphasises the CCF notion on interdependencies of capitals and the spiralling effect they have on each other.

Participation in CBPP strengthened community cohesion and trust. The community was able to coalesce and mobilise resources to construct health facilities such as Nhlupho rural health centre. The pooling of financial and material resources reflects a society that is united for a common cause.

One of the community leaders said ‘it is not easy to pool financial resources in this era to embark on such a huge project’. Not only is the community investing in infrastructure, community clubs have spread post the CBPP. The ability to contribute resources and have them kept within the area indicates the trust within the community. Community cohesion encourages local people to work in unity and helps them to bond to pursue common interests and goals. It is through this attribute that communities collectively reorganise themselves to confront diverse shocks and challenges bewildering them. The International Organization for Migration (IOM) Knowledge Management Hub (2020) in their community-based planning project that involved the construction of a community bridge learnt that collective efforts empower societies to lead their own integration processes by bringing the wider community together regardless of their differences. The CBPP was identified as a process that allowed communities to jointly reflect, analyse the context and identify precise needs and perspectives of others and worked collectively to improve everyone’s challenge. This process strengthened community belonging and made individuals gel strongly as they identified themselves as a joint group with a common goal. The intra and inter networks in a community improve as personal benefits in joining a network accrue through horizontal and vertical relations. In resonance, Daykin et al. (2021) opines that participatory approaches deepen connections offer opportunities for collectivism for coping with local and external shocks.

Demireva (2019) posits that despite the lack of standard measurement criteria of social cohesion and/or community gelling, community cohesion can be reflected in high levels of trust between individuals and the observance of common social norms. Some traditional measures of social capital such as being a member in community groups, associations and participation may reflect on trust established among community members. Women have formed village, savings and lending groups with the intent of raising household savings while others formed groups to improve the health infrastructure: an indication that a culture of working together has improved. Trust is also associated with communal responsibility where some previously marginalised groups are now trusted to assume important communal positions and are involved in decision-making. This study indicates that social capital is a vital factor for community success and a lack of social capital might spiral the community down with the opposite spiralling up (Mueller et al. 2020).

## Conclusion

The rollout of CBPPs as a resilience-planning tool invigorated collective actions and strengthened social networks that communities exploited to mobilise financial resources to set up a rural health centre and leveraged on their skills and other locally available materials. This makes the engagement of different socioeconomic groups imperative in shaping and making the prospects for sustainable sharing of ideas, experiences and benefits of interdependencies of community capitals a reality. Furthermore, CBPP processes are very instrumental in making communities collectively profile their vulnerabilities, devise gender sensitive strategies in dealing with recurrent shocks and note opportunities that may arise in different seasons owing to intra and inter networks. Gender issues cannot be ignored as they shape development needs and influence linkages accruing to men and women in societies and aid the design of context-specific interventions. Thus, inclusive and participatory processes have the propensity to strengthen community cohesion and trust as well as enhance the scope to collectively pull together. The ability of communities to pool resources contributes to their absorptive and adaptive capacities, which is central in building resilience. The working together of different socioeconomic groups accentuates the value of interdependencies of capitals and the spiralling effect they have on each other in leveraging resources for resilience building. Social capital is not the only desired capital for the coming together of communities; however, the need for other supportive capitals in local development generates a pull for collective actions.

## Footnotes

1 Painted dogs, hyenas and leopards are occasionally seen especially if they have left the National Park boundaries. Baboons normally cause some havoc during some farming seasons.

2 Infrastructure improvement, improved community cohesion, women empowerment, improved business structure, adoption of improved farming methods, improved participation in meetings, involvement of men, marginalised group involvement, increased income generation, diversified farming and improved working relationships.

3 Improved business and models, improved community inclusiveness and/or cohesion, women empowerment and decision making, diversified farming practices and improved social networks.
